# The Hen or the Egg: Inflammatory Aspects of Murine MPN Models

**DOI:** 10.1155/2015/101987

**Published:** 2015-10-12

**Authors:** Jonas S. Jutzi, Heike L. Pahl

**Affiliations:** ^1^Division of Molecular Hematology, University Hospital Freiburg, Center for Clinical Research, Breisacher Straße 66, 79106 Freiburg, Germany; ^2^Spemann Graduate School of Biology and Medicine (SGBM), University of Freiburg, Albertstraße 19A, 79104 Freiburg, Germany; ^3^Faculty of Biology, University of Freiburg, Schänzlestraße 1, 79104 Freiburg, Germany

## Abstract

It has been known for some time that solid tumors, especially gastrointestinal tumors, can arise on the basis of chronic inflammation. However, the role of inflammation in the genesis of hematological malignancies has not been extensively studied. Recent evidence clearly shows that changes in the bone marrow niche can suffice to induce myeloid diseases. Nonetheless, while it has been demonstrated that myeloproliferative neoplasms (MPN) are associated with a proinflammatory state, it is not clear whether inflammatory processes contribute to the induction or maintenance of MPN. More provocatively stated: which comes first, the hen or the egg, inflammation or MPN? In other words, can chronic inflammation itself trigger an MPN? In this review, we will describe the evidence supporting a role for inflammation in initiating and promoting MPN development. Furthermore, we will compare and contrast the data obtained in gastrointestinal tumors with observations in MPN patients and models, pointing out the opportunities provided by novel murine MPN models to address fundamental questions regarding the role of inflammatory stimuli in the molecular pathogenesis of MPN.

## 1. Introduction

“Dass Carcinome nicht selten auf einfach entzündliche Reize, wie Traumen, entstehen, ist bekannt” (that carcinomas arise, not seldom, at the site of inflammatory stimuli, such as traumas, is known) wrote Virchow in 1869 [1]. This far-sighted statement, worded as a fact rather than a hypothesis, was validated almost 150 years later when Hanahan and Weinberg named “inflammation” as an underlying principle that contributes to and fosters the newly named “hallmarks of cancer” [2].

## 2. Inflammatory Etiology of Solid Tumors

In the interval between these two pivotal publications, a large collection of data was accrued that supports the postulated role for inflammation in carcinogenesis. It is now known that solid tumors can arise on the basis of chronic inflammation, most notably Gastrointestinal Stromal Tumor (GIST) following* Helicobacter pylori* infection. Additional examples include enteropathy-associated T cell lymphoma and adenocarcinomas in patients with coeliac disease as well as the increased risk of colorectal carcinoma in patients with inflammatory bowel disease [3, 4].

**Figure 1:**
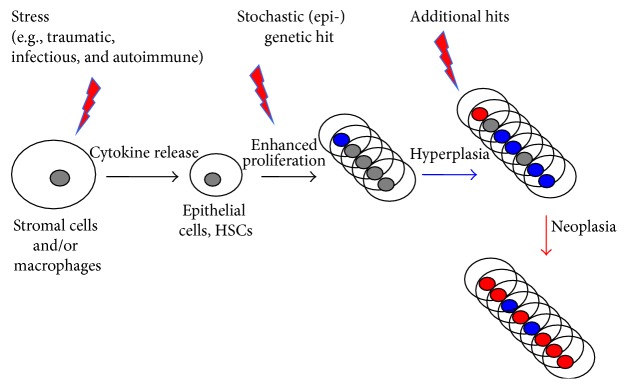
Multistep process for inflammatory driven neoplastic transformation. Stress, induced by various intrinsic and extrinsic factors, causes epithelial cells as well as stromal macrophages to release cytokines and other proliferation-promoting molecules, which lead to enhanced proliferation of surrounding cells. In a second step, enhanced proliferation increases the chance of stochastic mutations, leading first to hyperplasia and subsequently, with the accumulation of additional aberrations, to neoplasia.

The model for neoplastic transformation in these disorders implies a multistep process (Figure 1). Initially, chronic inflammation causes epithelial cells as well as stromal macrophages to release cytokines and other stimulatory molecules that promote proliferation of surrounding cells, for example, the interstitial cells of Cajal in the stomach during active* H. pylori* infection [5]. In a second series of steps, enhanced proliferation increases the chance of stochastic mutations, leading first to hyperplasia and subsequently, with the accumulation of additional aberrations, to neoplasia. While this model has been validated experimentally for several solid tumor entities, the role of inflammation in the genesis of hematological malignancies has not been extensively studied.

## 3. Cell Extrinsic Influences on the Development of Myeloid Malignancies

The microenvironment and stromal tissue that surround solid tumors can be seen as analogous in function and in cell-cell interactions to the bone marrow niche cells that surround hematopoietic stem cells. During the past years, several observations have strengthened the hypothesis that the bone marrow niche can contribute to the development of myeloid malignancies. In one seminal study, Raaijmakers and colleagues demonstrated that altering gene expression by deletion of* Dicer1* specifically in osteoprogenitor cells, but not in the bone marrow, led first to the development of myelodysplasia and, subsequently, to the emergence of acute myeloid leukemia [6]. Leukemia arose in hematopoietic cells that expressed* Dicer1* but had acquired other genetic abnormalities. Importantly, transplantation of BM from anemic, thrombocytopenic mice, in which* Dicer1* has been deleted in the osteoprogenitors, into lethally irradiated wild-type recipient mice led to complete resolution of the cytopenias, demonstrating that they were niche-induced and not attributed to cell autonomous changes in hematopoietic stem cells themselves [6]. Conversely, transplanting wild-type bone marrow cells into mice which carried the* Dicer1* deletion in osteoprogenitors resulted in an MDS phenotype and induction of AML. These data clearly demonstrate that changes in the bone marrow niche can be sufficient to induce leukemia. Interestingly, deleting* Dicer1* in mature osteoblasts did not induce either MDS or leukemia, demonstrating that very specific alterations in the bone marrow are required for niche-induced oncogenesis. The precise nature of these changes is currently being investigated and it is not known whether inflammatory mechanisms contribute to leukemia induction in this model.

## 4. Association of MPN with Inflammatory and Autoimmune Diseases

While the data by Raaijmakers and colleagues thus constitute a proof of principle that leukemia can be induced by changes in the bone marrow microenvironment, the question remains whether inflammatory processes in particular contribute to the induction or maintenance of myeloid malignancies, specifically to myeloproliferative neoplasms (MPN). Several studies have recently suggested an inflammatory etiology for MDS, AML, and MPN [7–10], most notably a large epidemiological study in Sweden, which demonstrated a significantly increased risk of AML or MDS in patients with a history of any infectious disease [9]. Esplin et al. have shown that continuous TLR activation by chronic exposure to Lipopolysaccharides (LPS) alters the self-renewal capacity of HSCs in mice. Prolonged TLR activation occurs in various bacterial infections, for example, during oral infections such as Gram-negative periodontitis and during subacute bacterial endocarditis [11]. In their mice, Esplin and colleagues were able to show a myeloid bias and, conversely, a selective loss of lymphopoietic potential as well as an increased proportion of CD150^hi^CD48^−^ long-term HSCs [12]. The emergence of a myeloid bias has been witnessed during normal aging of HSC [13–15]. Signer et al. point out that the risk of developing myeloid and lymphoid leukemias increases with age [16]. It seems likely that HSCs acquire random genetic hits either under chronic TLR activation induced by LPS or during normal aging. These parallels strengthen the hypothesis of inflammatory driven myeloid malignancies, in some cases perhaps induced directly by an infectious cause.

While inflammatory processes involve various factors, including cytokines, reactive oxygen species, and immune cells like macrophages, autoimmune phenomena are characterized by activation of T and B cells including the production of autoantibodies. Autoimmune diseases thus mainly involve changed T and B cell function but might share aspects of inflammatory processes resulting from altered cytokine release, such as increased IL-6 levels [17].

MPN patients with an antecedent autoimmune disorder carried a 1.7- and 2.1-fold increased risk to develop an AML or an MDS, respectively [9, 18]. In particular patients with MPN-associated myelofibrosis may show various autoimmune phenomena, including antibodies against red blood cells or anti-nuclear [9, 18] or anti-mitochondrial antibodies. To some extent, this might explain the pathogenesis of anemia and the accompanied compensatory reticulocytosis in this cohort of patients [19, 20]. The resulting increased malignant and nonmalignant myeloproliferation themselves thereby increase the risk for stochastic secondary (epi-)genetic hits and disease progression. However, neither the inflammatory nor the autoimmune hypotheses regarding MPN etiology have yet been directly confirmed by experimental studies.

## 5. The Inflammatory Hypothesis of MPN

MPN patients show elevated serum levels of various proinflammatory cytokines including IL-1, IL-6, IL-8, IL-11, IL-17, TNF-*α*, and TGF-*β*, as well as of the anti-inflammatory IL-10 [21–26]. Treatment with Ruxolitinib, JAK1 and JAK2 inhibitor, significantly decreased the level of circulating cytokines [27]. While these data demonstrate that MPN is accompanied by inflammatory changes, the causal order of events has not been determined. Does the malignant clone trigger an inflammatory response or—and this would constitute a change in perspective—can chronic inflammation itself trigger a MPN? In the latter model, sustained low-level, probably subclinical inflammation initially increases the proliferation of healthy, polyclonal hematopoietic stem and progenitor cells. Since each cell division carries the risk of acquiring a mutation, a malignant MPN clone arises and evolves on the basis of chronic, inflammation-induced proliferation.

Is there evidence supporting such a change in perspective or can it be procured using recently established, novel murine MPN models?

## 6. Murine Models to Test the Inflammatory Hypothesis of MPN

The field of gastrointestinal tumors has made use of sophisticated mouse models to detail the role of inflammation for the initiation and promotion of carcinomas. Multiple tissue specific knockout and transgenic lines have been generated to study the underlying molecular mechanisms and signal transduction pathways [34]. During the past five years, various mouse models with a myeloproliferative neoplasm- (MPN-) like phenotype have also been reported [32, 33, 45–52]. In this review, we will describe the evidence supporting a role for inflammation in initiating and promoting MPN development. Furthermore, we will compare and contrast the data from GI tumors with observations in MPN patients and models, pointing out the opportunities provided by the novel murine MPN models to address fundamental questions regarding the role of inflammatory stimuli in the molecular pathogenesis of MPN.

Various murine MPN models based on the most commonly occurring mutations have been developed. The alleles, which were introduced either in bone marrow transplant models, as transgenes, or as constitutively or inducibly active knock-ins, include JAK2^V617F^, JAK2^Exon12^, cMpl^W515L^, TET2, ASXL1, and NFE2 (see Table 1) [32, 33, 45–52]. Of these, the NFE2 mice consistently show spontaneous transformation to acute leukemia, suggesting that elevated NFE2 activity promotes not only MPN development but also a sustained acquisition of additional aberrations leading to leukemic transformation [32, 33]. The transcription factor NFE2 is overexpressed in the majority of MPN patients, irrespective of the underlying driver mutation [53, 54]. NFE2 is central to the inflammatory process. On the one hand, it is induced by inflammatory cytokines, such as IL1*β* [55]. Elevated NFE2 activity in turn increases cell proliferation by increasing transcription of cell cycle regulators and promoting G1/S transition [33]. On the other hand, NFE2 itself promotes inflammation as it has been shown to directly regulate transcription of IL-8, a proinflammatory cytokine [56]. Interestingly, inhibition of NFE2, by shRNA, abrogates endogenous erythroid colonies (EEC) formation [57], a pathognomonic hallmark of PV, supporting a central role for this inflammatory axis in promoting growth of the neoplastic clone.

Two distinct groups of murine models are used to study the role of inflammation in GI cancers (reviewed in [34]). The first are genetically altered mice, either transgenic or knock-in strains, that carry mutations in the “adenomatous polyposis coli” (APC) gene or in genes affecting the Wnt signaling pathway. The APC gene is mutated in 80% of human colorectal cancers, while a further 10% carry mutations in beta-catenin, a central regulator of the Wnt-signaling pathway [58, 59]. In the second type of models, chemical carcinogens and promoters of inflammation, frequently azoxymethane (AOM) and dextran sodium sulfate (DSS), are used to induce the development of colitis associated colon cancer (CAC) [34].

## 7. The Role of the COX2/PGE2 Axis

By generating double or triple mutant mice, for example, strains that carry APC mutations in addition to tissue specific knockouts of critical signal transducing molecules, the role of various molecular pathways was investigated. The data reveal a critical role for the cyclooxygenase-2 (COX-2)/prostaglandin-E2 (PGE2) pathway even in mice that carry APC mutations [35–37, 43]. COX-2 is a central mediator of inflammation. It oxidizes arachidonic acid to prostaglandin H2, which is subsequently converted to PGE2. PGE2 promotes inflammation by affecting a variety of cellular functions. In contrast to COX-1, which is constitutively expressed, COX-2 is specifically induced by proinflammatory stimuli and mitogens.

Knockout of COX-2 in mice carrying the APC^Δ716^ mutation drastically suppressed the development of intestinal polyposis as did treatment of mice with COX-2 inhibitors [35]. Conversely, transgenic overexpression of COX-2 in colon epithelium increased the development of intestinal tumors [43]. A similar strategy could easily be used to test the importance of the COX-2/PGE2 axis in MPN models. The COX-2 knockout is not tissue specific, so that development of the MPN phenotype in the presence or absence of systemic COX-2 could be investigated. In this context, the use of inducible models appears especially interesting, as the role of inflammatory processes in disease initiation could be investigated [48, 50, 60].

The logic described above was applied to various other genes in the COX-2/PGE2 axis, and the results consistently underwrite an essential role for an inflammatory response in the development of APC-driven cancers. For example, knockout of the gene for either the PGE2-receptor-2 or the microsomal PGE synthase resulted in the suppression of intestinal polyp formation [37]. Conversely, deletion of the gene for 15-prostaglandin dehydrogenase (15-PDGH), an enzyme that catabolizes and inactivates prostaglandins, resulted in disease exacerbation, animals carrying mutant APC but lacking 15-PDGH developing significantly more polyps than their control littermates [38]. In addition, and perhaps less surprisingly, the COX-2/PGE2 axis was also shown to be essential in the AOM/DSS inflammation-associated colon tumor model, as deletion of COX-2 exacerbates CAC development [44, 61].

Equivalent mouse strains could be generated in the context of various MPN mutations to investigate the contribution of the COX-2/PGE2 inflammatory axis to MPN disease initiation or maintenance. Inducible expression of MPN alleles in the background of a constitutive COX-2/PGE2 knockout will test the role of inflammation in MPN initiation, whereas constitutive expression of MPN mutations and subsequent inducible deletion of a COX-2/PGE2 axis gene will test for the requirement of an inflammatory milieu in maintaining the MPN phenotype.

## 8. The Role of Specific Immune Cells

During the past decade, various mouse strains lacking specific immune cells have been developed. These mice can attest to the requirement of specific cell types for disease development. For example, crossing APC^Δ716^ mice with op/op mice, which are devoid of functional macrophages, led to a suppression of polyp formation, as did the generation of APC-mutant, kit^W/W^ mice, which lack mast cells [62]. Hence, both macrophages and mast cells are required to elaborate the microenvironment in which mutant APC can induce polyp formation. A recent paper by Ramos and colleagues provides compelling evidence that similar but distinct mechanisms operate in MPN [63]. In mice with an established JAK2^V617F^ driven erythrocytosis, depletion of macrophages with clodronate normalized hematocrit and RBC counts as well as reducing reticulocytosis. Since these authors used a Vav-Cre/JAK2^V617F^ BMT model, it is likely that the macrophages were also carrying the JAK2^V617F^ mutation and were therefore part of the malignant clone. The molecular mechanism is thus slightly different from that in gastric cancer, where macrophages appear necessary for paracrine stimulation of the neoplastic epithelial cells. In MPN, macrophages that are part of the malignant clone would be perpetuating the neoplasia in an autocrine manner. However, if the op/op mice are used in models similar to those detailed above, a role for healthy macrophages in MPN initiation from healthy HSCs may be revealed.

## 9. The Role of Cytokines

The requirement for macrophages and mast cells points to a rather obvious role for cytokines in tumor formation. While the essential role for cytokines in various physiological processes makes the construction of knockout mice deficient in these signaling molecules challenging, several strains have been generated and examined for cytokine contribution to gastric cancer development. Deletion of IL-17, IL-6, CCR2, or TNF-receptor p55 [39–42] led to a suppression of intestinal polyp development or CAC development in both the APC-mutant and the AOM/DSS models.

One very similar study points to an important role for TNF-*α* in promoting JAK2^V617F^ driven MPN [28]. Deletion of TNF-*α* limited expansion of JAK2^V617F^ positive cells and attenuated disease development, pointing to a disease-promoting role for this cytokine. Analogous investigations for other inflammatory cytokines are required, especially addressing the question whether they are necessary for successful disease initiation. Candidates that should be investigated with priority include those factors for whom elevated levels have been documented in MPN patients and who have been shown to play a role in the genesis of other entities with an inflammatory component.

In this light, IL-11 stands out as its levels are elevated in PV patients and has been shown to induce healthy bone marrow to form endogenous erythroid colonies [22, 64]. EEC constitute a characteristic abnormality of PV, one that may be used diagnostically because of its high sensitivity and specificity. Antibodies to IL-11 inhibit EEC formation in PV cells [64]. IL-11 has been shown to promote gastric tumor development, while, conversely, deletion of the IL-11 coreceptor alpha ablated the development of gastric tumors [65].

IL-8 has likewise been shown to induce EEC formation from healthy bone marrow cells [64]. As detailed above, IL-8 is a direct target of NFE2 and both are overexpressed in MPN patients. Furthermore, Hermouet and colleagues have shown that IL-8 promotes hematopoietic progenitor survival [66]. Conversely, inactivation of the IL-8 pathway inhibited CD34^+^ cell proliferation and colony formation [66]. As IL-8 levels constitute an independent predictor of survival in PMF patients, this cytokine is highly likely to contribute to MPN pathophysiology, perhaps as one of the pivotal inflammatory mediators that initiate hyperproliferation of healthy HSCs in the bone marrow [26].

The role of TGF-beta in the dysmegakaryopoiesis and fibrosis characteristic of PMF has been investigated in a murine model of myelofibrosis due to low Gata-1 expression (Gata-1^lo^) [29, 30]. While the mutation decreasing Gata-1 levels in this model is not found in PMF patients, Gata-1 levels are specifically downregulated in a subset of PMF megakaryocytes [67]. In Gata-1^lo^ mice, inhibition of TGF-beta signaling restored hematopoiesis, normalized megakaryocyte development, and reduced fibrosis [30]. Similar results were obtained by Dr. Vainchenker's group in mice overexpressing thrombopoietin (TPO). Mice displaying high TPO levels develop an MPN phenotype with fibrosis. In the absence of TGF-beta, these mice still show a myeloproliferative syndrome, yet no fibrosis [31]. Interestingly, while they express normal TGF-beta levels, untreated Gata-1^lo^ mice nonetheless show specific TGF-beta signaling alterations in bone marrow and spleen, such as overexpression of EVI1. This signaling abnormality is comparable to the abnormal TGF-beta profile observed in PMF patients, which includes overexpression of STAT1 and IL-6, factors directly related to autoimmune fibrosis [68].

These data clearly indicate that TGF-beta plays a pivotal role in propagating the PMF phenotype and the development of fibrosis, which contributes to the cytopenias that constitute the leading cause of morbidity and mortality in this patient population. Targeted deletion or tissue specific overexpression of TGF-beta is now required to determine whether the cytokine is required or sufficient for disease initiation. Observations in other organs suggest that the latter is likely: liver specific overexpression of TGF-beta results in hepatic fibrosis [69].

Another novel, autocrine inflammatory pathway has recently been described. Dr. Hoffman's laboratory showed that MPN myeloid cells secrete elevated levels of lipocalin-2, an inflammatory cytokine, and that lipocalin-2 levels are elevated in PMF patients [70]. Lipocalin secretion is known to be stimulated by IL-1, IL-6, and IL-17, all of which are elevated in MPN [23, 24, 71–73]. Lipocalin induces reactive oxygen intermediates (ROS) formation with subsequent induction of double stranded DNA breaks leading to apoptosis of healthy HSCs but not PMF HCSs [70]. Hence, protection of PMF cells from lipocalin action, by a yet unknown mechanism, could constitute one way in which the microenvironment or the MPN clone itself uses inflammatory mediators to create an environment that provides a selective advantage to the MPN clone.

## 10. The Inflammatory Hypothesis of MPN: Awaiting Proof from Murine Models

While the evidence presented above supports a change in perspective, in which inflammation may induce and promote MPN, rather than simply being a consequence of it, several aspects of this hypothesis remain to be experimentally proven. A murine model, which does not carry a specific MPN mutation, but rather models a prolonged, chronic inflammation, would constitute a valuable tool. If in such a model, the inflammatory milieu alone was sufficient to induce malignant myeloproliferation or even leukemic transformation, this would constitute a proof of principle.

Proving the inflammatory hypothesis in MPN patients directly may, however, not be feasible. Diagnosing the underlying inflammatory process, postulated to be present even prior to the clinical MPN presentation, will not be possible in most cases. However, this will not be required. If the inflammatory hypothesis can be proven experimentally, this provides sufficient evidence for the initiation of clinical trials examining the effectiveness of early therapeutic intervention with the goal of suppressing chronic inflammation, thereby intersecting the vicious cycle that promotes MPN progression. Again, epidemiological data from the field of gastric cancers may point the way. Two landmark studies, published over 20 years ago, demonstrated that regular use of nonsteroidal anti-inflammatory drugs (NSAIDs) reduces the risk of colon cancer [74]. NSAIDs including aspirin are well known to function as a COX-1/2 inhibitor and therefore inhibit the production of PGE2. The Efficacy and Safety of Low-Dose Aspirin study (ECLAP), nomen est omen, proved both the safety and efficacy of aspirin in PV patients [75]. While overall survival was not increased during the observation period in patients treated with low-dose aspirin, longer followup is required to observe a beneficial effect if aspirin use prevents leukemic transformation by suppressing a chronic inflammatory stimulus. As mentioned above, mouse strains carrying MPN mutations in the context of COX-2 deficiency may reveal the impact of the COX-2/PGE2 inflammatory axis to MPN disease initiation and maintenance as well as leukemic progression.

## Figures and Tables

**Table 1:** Disease models involving inflammation.

Affected compartment	Cause	Intervention	Phenotype	Reference
	Genetic alteration			

Hematopoiesis	JAK2^V617F^	TNF-*α* deletion	Attenuation of MPN development	[28]
Hematopoiesis	Gata-1^lo^		Myelofibrosis	[29]
Hematopoiesis	Gata-1^lo^	TGF-*β* inhibition	Restored hematopoiesis, reduced fibrosis	[30]
Hematopoiesis	TPO^hi^ with	TGF-*β* inhibition	Restored hematopoiesis	[31]
Hematopoiesis	NFE2 overexpression/mutations		MPN, sAML	[32, 33]
Gastrointestinal mucosa	APC mutations		Colorectal cancer	Reviewed in [34]
Gastrointestinal mucosa	APC^Δ716^	COX-2 knockout	Suppression of intestinal polyposis	[35]
Gastrointestinal mucosa	APC^Δ716^	PGE2-receptor-2 knockout	Suppression of intestinal polyposis	[36]
Gastrointestinal mucosa	APC^Δ716^	Prostaglandin synthase knockout	Suppression of intestinal polyposis	[37]
Gastrointestinal mucosa	APC^Δ716^	15-prostaglandin dehydrogenase (15-PDGH) knockout	Disease exacerbation	[38]
Gastrointestinal mucosa	APC^Δ716^	Deletion of either IL-17, IL-6, CCR2, TNFR, or p55	Suppression of intestinal polyposis	[39–42]

	Infectious cause			

Hematopoiesis cell intrinsic and extrinsic	TLR activation by bacterial infection		HSC exhaustion	[12]

	Chemical cause			

Gastrointestinal mucosa	Azoxymethane (AOM) Dextran Sodium Sulfate (DSS)		Colitis associated colon cancer (CAC)	Reviewed in [34]
Gastrointestinal mucosa	Azoxymethane (AOM)	COX-2 transgene	Increased development of tumors	[43]
Gastrointestinal mucosa	Azoxymethane (AOM) Dextran Sodium Sulfate (DSS)	COX-2 deletion	Increased development of tumors	[44]
Gastrointestinal mucosa	AOM or DSS plus deletion of either IL-17, IL-6, CCR2, TNFR, or p55		Suppression of CAC	[39–42]
